# Calcar screws and adequate reduction reduced the risk of fixation failure in proximal humeral fractures treated with a locking plate: 190 patients followed for a mean of 3 years

**DOI:** 10.1186/s13018-018-0906-y

**Published:** 2018-08-09

**Authors:** Sjur Oppebøen, Annette K. B. Wikerøy, Hendrik F. S. Fuglesang, Filip C. Dolatowski, Per-Henrik Randsborg

**Affiliations:** 1 Department of Orthopaedic Surgery, Akershus University Hospital, Lørenskog, Norway; 2 Faculty of Medicine, University of Oslo, Oslo, Norway

**Keywords:** Proximal humeral fracture, Locking plate fixation, Fixation failure, Calcar screws, Reoperation, Long-term shoulder function

## Abstract

**Background:**

Fixation of proximal humeral fractures (PHF) with locking plates has gained popularity over conservative treatment, but surgery may be complicated with infection, non-union, avascular necrosis (AVN) of the humeral head and fixation failure. Failure to achieve structural support of the medial column has been suggested to be an important risk factor for fixation failure. The aims of this study were to examine the effect of calcar screws and fracture reduction on the risk of fixation failure and to assess long-term shoulder pain and function.

**Methods:**

This was a single-centre retrospective study of 190 adult PHF patients treated with a locking plate between 2011 and 2014. Reoperations due to fixation failure were the primary outcome. Risk factors for fixation failure were assessed using the Cox regression analysis. Postoperative shoulder pain and function were assessed by the Oxford Shoulder Score (OSS).

**Results:**

Thirty-one of 190 (16%) patients underwent a reoperation: 14 (7%) due to fixation failure, 10 (5%) due to deep infection and 2 (1%) due to AVN. The absence of calcar screws and fixation with residual varus malalignment (head-shaft angle < 120°) both increased the risk of fixation failure with an adjusted hazard ratio (95% CI) of 8.6 (1.9–39.3; *p* = 0.005) and 4.9 (1.3–17.9; *p* = 0.02), respectively. The median (interquartile range) OSS was 40 (27–46).

**Conclusion:**

The use of calcar screws, as well as the absence of postoperative varus malalignment, significantly reduced the risk of fixation failure. We, therefore, recommend the use of calcar screws and to avoid residual varus malalignment to improve the medial support of proximal humeral fractures treated with a locking plate.

## Background

Proximal humeral fractures (PHFs) represent the third most frequent fragility fracture [1]. The incidence of surgical treatment has increased, and fracture fixation with locking plates has become a popular treatment [2]. Cochrane reviews do not support this trend [3]. Patients treated surgically probably need more subsequent surgery than conservatively treated patients [4]. Reoperation rates after primary fixation of PHFs may reach 30% [5] due to mechanical fixation failure, surgical site infection, screw perforation or avascular necrosis (AVN) of the humeral head. Failure to achieve structural support of the medial column has been suggested to be an important risk factor for fixation failure [6–9].

We, therefore, studied the effect of calcar screws and postoperative varus malalignment on the risk of fixation failure in adult patients with a PHF treated with a locking plate. A secondary aim was to assess long-term shoulder pain and function reported by these patients.

## Methods

The patients were retrospectively identified by a digital search for locking plate fixations of PHF performed at Akershus University Hospital, Norway, between January 2011 and December 2014. We identified 229 eligible adult patients (18 years and above). As explained in Table 1, 39 (17%) patients were not included, and we did not send the OSS questionnaire to 43 patients.

**Table 1:** Reasons for patient exclusion. 190 patients were included for final analysis

Excluded from all outcomes (39 patients)	Not invited to complete OSS (43 patients)
• Primary treatment delayed more than 3 weeks (15 patients)	• Deceased (23 patients)
• Surgery performed due to non-union following conservative treatment (8 patients)	• Considered non-compliant (dementia/alcoholism/drug abuse) (11 patients)
• Isolated fracture of the greater tuberosity (5 patients)	• A concomitant fracture in the same upper extremity (4 patients)
• Neurovascular injury at presentation (2 patients)	• Patients with pre-existing shoulder complaints (3 patients)
• Open fracture (2 patients)	• Did not speak Norwegian (2 patients)
• Other reasons (7 patients)	

For the final analyses, 190 patients were included. The patients’ medical records were reviewed for patient, fracture and procedural characteristics. Furthermore, we recorded clinical and radiological results including surgically related complications and reoperations. Patients were retrospectively observed until whichever of the following events first occurred: re-operation, end of study period or death. The individual times to events were used in the Cox regression analyses, and the end of the study period was July 30, 2016, for all patients.

**Fig. 1:**
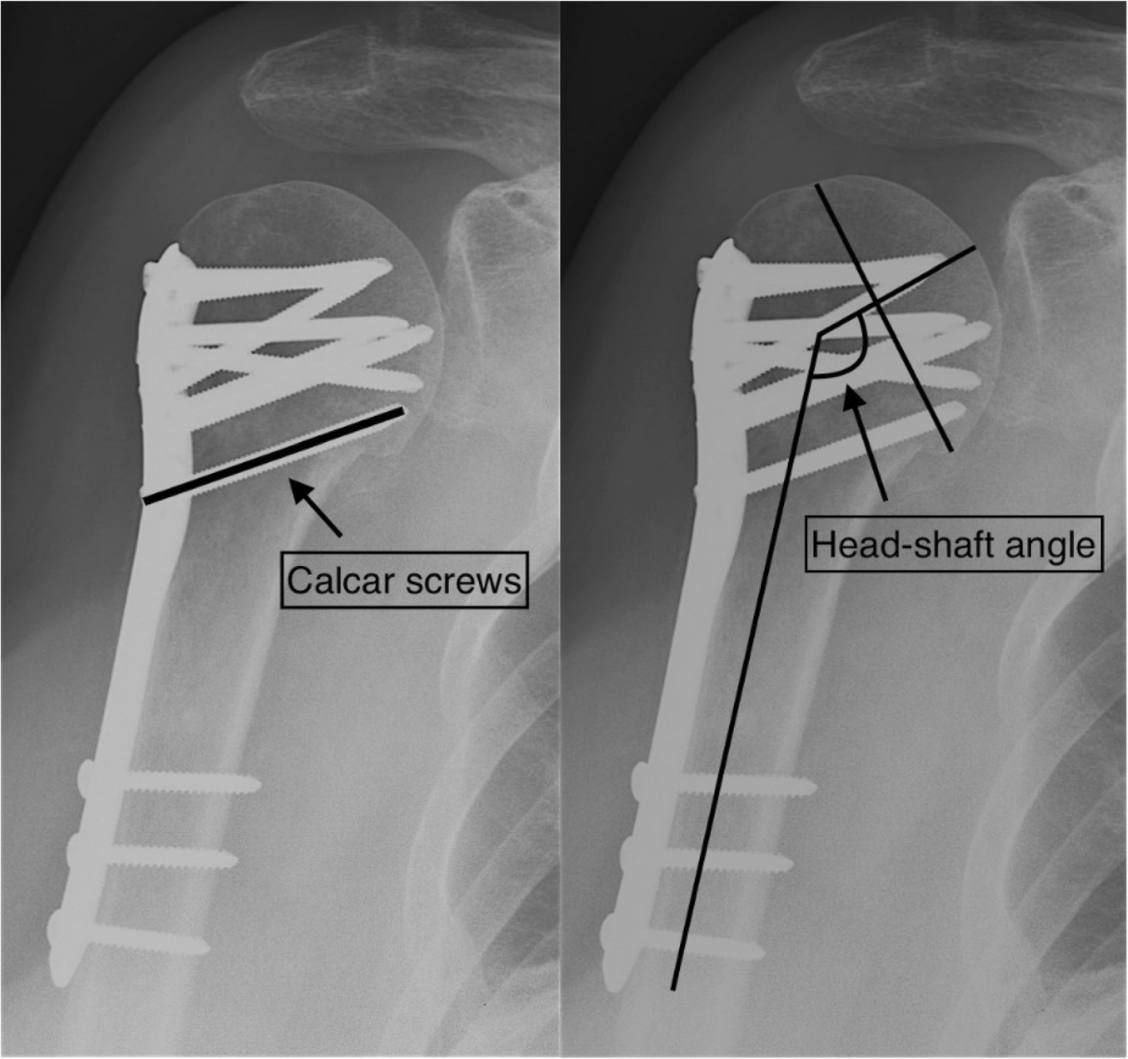
Correct positioning of the calcar screws with purchase in the inferiomedial quadrant of the humeral head (left). Measurement of the head-shaft angle (HSA). The HSA is the angle created between a line perpendicular to the anatomical neck plane and the axis of the humerus shaft (right)

Two of the authors (SO and AKBW), blinded for the clinical outcome, assessed pre- and post-operative anteroposterior (AP) and trans-scapular shoulder radiographs stored in the hospital’s digital image database. Patients were categorised in two groups by the presence or absence of calcar screws as seen in postoperative radiographs (Fig. 1). Calcar screws were defined as obliquely placed 3.5 mm screws locked to the plate and with purchase in the inferomedial quadrant of the humeral head [10]. Pre and postoperative fracture malalignment was evaluated by measuring the head-shaft angle (HSA) using the method described by Agel [11] (Fig. 1). Consistent with previously published data, postoperative varus malalignment was defined as an HSA < 120° [12] and an HSA of 135° defined the physiological reference value [13]. Fractures were categorised according to the number of fragments as two, three or four part fractures, corresponding to the Arbeitsgemeinschaft für osteosynthesefragen/Orthopaedic Trauma Association’s (AO/OTA) classification A, B or C type fractures, respectively [14].

Patient-reported shoulder pain and function were assessed by a validated Norwegian version of the Oxford Shoulder Score (OSS), consisting of 12 questions with scores ranging from 0 (worst possible result) to 4 (best possible result) [15]. OSS was graded as poor (< 36 points), good (36–41 points) and excellent (> 41 points) [16]. The OSS questionnaire was mailed to 147 of 190 (77%) patients during June and July, 2016, and 43 patients (23%) were not eligible to complete the questionnaire (Table 1).

During the study period, our centre considered patients with the following fracture characteristics for open reduction and locking plate fixation: an HSA < 105° (varus displacement), an HSA > 180° (valgus displacement) and displacement of the tuberosities more than 5 mm and/or less than 50% contact between the shaft and the head fragments. Patients who were offered locking plate fixation, received general anaesthesia, an inter-scalene brachial plexus block, or both, and were positioned in a beach chair position. Open reduction was performed through either a classic deltopectoral approach or by the less invasive deltoid split approach, depending on fracture characteristics and surgeon’s preference. The surgeon reduced the fracture under fluoroscopic guidance by the aid of non-absorbable sutures placed in the rotator cuff and temporary fixation with Kirschner wires. A 5-hole locking plate (PHILOS, DePuy Synthes, West Chester, Pennsylvania, USA) was temporarily fixed to the reduced fracture fragments using Kirschner wires and the cuff sutures and approximated to the humeral shaft using a 3.5 mm cortical screw. If a deltoid split approach was used, the shaft screws were placed percutaneously or through a mini-open approach. Finally, 3.5 mm locking screws were inserted into the humeral head after subtracting 5 mm screw length from the joint line contour to minimise the risk of subsequent perforation. No patients received medial buttress plate or allograft reinforcement during the study period. Bone substitute (HydroSet, Stryker, or STRUCSURE CP, Smith & Nephew) was used in valgus impacted fractures at the surgeon’s discretion*.* All patients received three doses of perioperative antibiotic prophylaxis (cloxacillin 2 g + 1 g + 1 g, or clindamycin 600 mg + 300 mg + 300 mg in case of penicillin allergy).

**Fig. 2:**
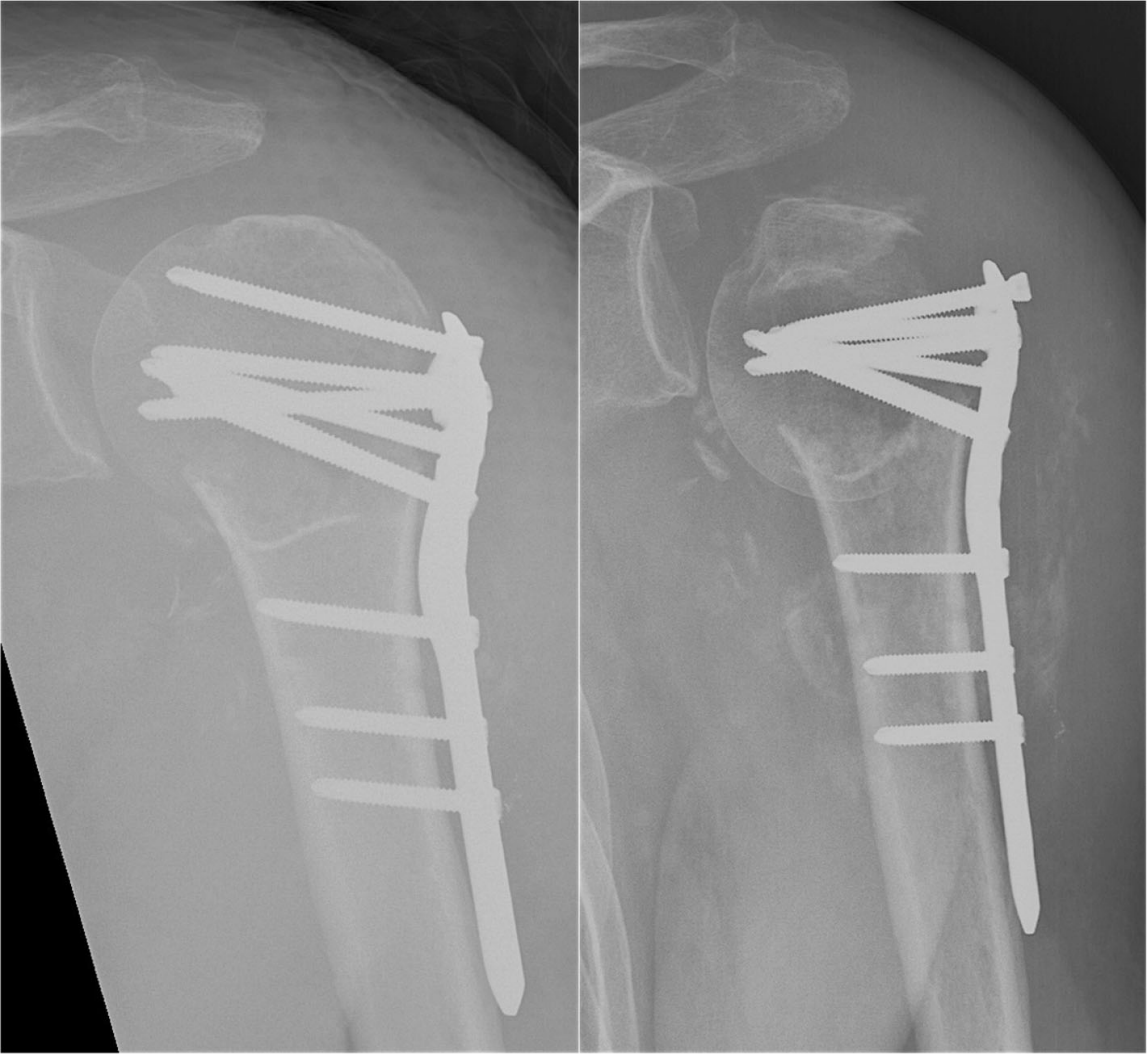
A proximal humeral fracture in a 68-year-old female treated with a locking plate without calcar screws (left). Failure of fixation with varus collapse 6 weeks after primary surgery (right)

Physiotherapists instructed all patients orally and in writing in post-operative rehabilitation exercises as per local guidelines. Patients were routinely scheduled for follow-up after 6–8 weeks (Fig. 2). Patients with prolonged pain, radiologically evident loss of reduction, fixation failure, non-union or AVN were scheduled for additional follow-ups. These patients were followed every 6–8 weeks with radiographs until union, or until they were scheduled for revision surgery with re-fixation or arthroplasty.

### Statistics

The Shapiro–Wilk test was used to assess continuous variables for normal distribution. Normally distributed variables were described by means and standard deviations (SDs) and non-normally distributed variables by medians and interquartile ranges (IQRs). The corresponding statistical tests used to compare differences between the two groups were independent two-sample *t* test and the Mann–Whitney U test. Categorical variables were described as numbers and percentages, and differences between groups were compared using z-statistics. Inter-rater reliability (IRR) for radiological measurements was assessed using the intraclass correlation coefficient (ICC). The Cox proportional hazards regression analysis was used to assess risk factors for fixation failure. The assumption of proportional hazards was tested by inspection of log-minus-log plots. In the multivariable Cox regression, the variables were adjusted for age and sex. Statistical analyses were performed using the IBM SPSS version 24 for Mac.

### Ethics

The regional ethical committee of south east Norway reviewed and delegated the approval of this study (reference no. 2015/1435) to the Data Protection Official at Akershus University Hospital who granted the ethics approval (reference no. 15–128).

## Results

Ninety-three of 190 (49%) patients were operated without calcar screws and 97 of 190 (51%) with calcar screws. The median follow-up time was 35 (range 0.3–65) months, and the median time for the last radiological examination was 3, 4 (range 0,03–53) months.

Patient demographics, fracture characteristics and perioperative circumstances, categorised by application of calcar screws are presented in Table 2.

**Table 2:** Patient, fracture and procedural characteristics in 190 patients with a proximal humeral fracture

	No calcar screws (*n* = 93)	Calcar screws (*n* = 97)	Mean difference or relative risk (95% CI)	*p* value
Age (years), mean (SD)	65 (15)	69 (13)	4.7 (0.7–8.7)	0.02 ^b^
median (IQR)	68 (17)	70 (20)	–	0.06 ^c^
Women	64 (69)	68 (70)	1.0 (0.8–1.2)	0.8 ^d^
Smokers	21 (23)	27 (28)	0.8 (0.5–1.3)	0.4 ^d^
Diabetes	13 (14)	8 (8)	1.7 (0.7–3.9)	0.2 ^d^
Non-compliance	9 (10)	7 (7)	1.3 (0.5–3.5)	0.5 ^d^
ASA III or IV	31 (33)	23 (24)	1.4 (0.9–2.2)	0.1 ^d^
3 or 4 part fracture type	68 (73)	74 (76)	1.0 (0.8–1.1)	0.6 ^d^
Fracture dislocation	7 (8)	6 (6)	1.2 (0.4–3.5)	0.7 ^d^
Preoperative varus < 105°	22 (24)	21 (22)	1.1 (0.6–1.8)	0.7 ^d^
Preoperative valgus > 180°	16 (17)	19 (20)	0.9 (0.5–1.6)	0.7 ^d^
Medial comminution	54 (58)	75 (77)	0.8 (0.6–0.9)	0.006 ^d^
Surgical approach: deltoid split	69 (74)	40 (41)	1.8 (1.4–2.3)	< 0.001 ^d^
Residual varus malalignment < 120° ^a^	6 (7)	5 (5)	1.3 (0.4–4.0)	0.7 ^d^

Percentages in parenthesis unless stated otherwise

*CI* confidence interval, *SD* standard deviation, *IQR* Interquartile range, *ASA* American Society of Anaesthesiologists

^a^*n* = 88 and 93, ^b^Independent two-sample *t* test,^c^ Mann–Whitney test, ^d^z statistics

Patients operated without calcar screws were nearly 5 years younger than those operated with calcar screws (mean difference = 4.7 (95% CI: 0.7–8.7); *p* = 0.02). Patients with an intact medial column of the proximal humerus were more often operated without calcar screws, and these fractures were more often approached by deltoid split. Patients with preoperative varus (HSA < 105°) were more often fixated in residual varus malalignment (HSA < 120°) compared to those who presented with a preoperative HSA > 105° (8 of 11 (73%) versus 33 of 170 (19%); *p* < 0.001). Thirty-one of 190 (16%) patients with PHF treated with a locking plate required a reoperation: 14 (7%) due to fixation failure, 10 (5%) due to deep surgical site infection and 2 (1%) due to avascular necrosis of the humeral head. In addition, 5 (3%) patients underwent hardware removal due to presumably implant-related local shoulder pain. Patients operated without calcar screws more often required a reoperation than those who received calcar screws (12 of 93 (13%) versus 2 of 97 (2%); *p* = 0.005) (Table 3).

**Table 3:** Reoperations in 190 patients with a proximal humeral fracture treated with a locking plate

Indication	No calcar screws (*n* = 93)	Calcar screws (*n* = 97)	Relative risk (95% CI)	*p* value
Fixation failure	12 (13)	2 (2)	6.3 (1.4–27.2)	0.01 ^a^
Deep infection	4 (4)	6 (6)	0.7 (0.2–2.4)	0.6 ^a^
Avascular necrosis	1 (1)	1 (1)	1.0 (0.06–16.4)	1.0 ^a^
Local pain	3 (3)	2 (2)	1.6 (0.3–9.2)	0.6 ^a^

Percentages in parenthesis

*CI* confidence interval

^a^z statistics

Patients operated without calcar screws had a 6-fold increased risk of a reoperation due to fixation failure compared to those who received calcar screws (hazard ratio (HR) = 6.5 (95% CI 1.5–28.9; *p* = 0.02) (Table 4).

**Table 4:** Risk factors for fixation failure. Univariate Cox regression analysis with time to fixation failure as outcome

Covariate	*n*	Hazard ratio	95% CI	*p* value
Age, increase of 1 year	69 ^b^	1.05	(1.0 to 1.1)	0.04
Sex				
Women	133	1 ^a^		
Men	57	0.59	(0.2 to 2.1)	0.4
Smoker				
No	142	1 ^a^		
Yes	48	0.80	(0.2 to 2.9)	0.7
Diabetes				
No	169	1 ^a^		
Yes	21	0.61	(0.1 to 4.7	0.6
Non-compliant				
No	173	1 ^a^		
Yes	17	5.35	(1.7 to 17.1)	0.005
ASA classification				
ASA I-II	136	1 ^a^		
ASA III-IV	54	2.7	(1.0 to 7.7)	0.06
Fracture classification				
2 part	48	1 ^a^		
3 or 4 part	142	2.1	(0.5 to 9.5)	0.3
Fracture dislocation				
No	177	1 ^a^		
Yes	13	2.3	(0.5 to 10.3)	0.3
Preoperative varus < 105°				
No	147	1 ^a^		
Yes	43	2.8	(1.0 to 8.0)	0.06
Preoperative valgus > 180°				
No	155	1 ^a^		
Yes	35	1.8	(0.6 to 5.7)	0.3
Medial comminution				
No	60	1 ^a^		
Yes	130	1.9	(0.5 to 6.6)	0.3
Surgical approach				
Deltopectoral	82	1 ^a^		
Deltoid split	108	0.5	(0.2 to 1.5)	0.3
Calcar screws				
Yes	97	1 ^a^		
No	93	6.5	(1.5 to 28.9)	0.02
Adequate reduction > 120°				
Yes	179	1 ^a^		
No (varus)	11	5.2	(1.5 to 18.7)	0.01

*CI* Confidence Interval, *ASA* American Society of Anaesthesiologists

^a^Reference category, ^b^median age

Adjusting for age and gender, the risk of fixation failure without the use of calcar screws was even greater (HR = 8.6 (95% CI 1.9–39.3; *p* = 0.005) (Table 5).

**Table 5:** Multivariate Cox proportional hazards analyses with time to fixation failure as outcome

Covariate	*n*	Hazard ratio	95% CI	*p* value
Calcar screws				
Yes	97	1 ^a^		
No	93	8.6	(1.9 to 39.3)	0.005
Adequate reduction > 120°				
Yes	179	1 ^a^		
No (residual varus)	11	4.9	(1.3 to 17.9)	0.02

^a^ Reference category

Fixation of the humeral head with a residual varus malalignment (HSA < 120°) also increased the risk of reoperations due to fixation failure (HR = 5.2 (95% CI 1.5–18.7; *p* = 0.01), and adjusting for age and gender, HR = 4.9 (95% CI 1.3–17.9; *p* = 0.02). The interrater agreement for measurements of the postoperative HSA was excellent (ICC (95% CI) = 0.91 (0.88–0.93) [17].

There were 132 of 148 (89%) patients who completed the OSS questionnaire. The median long-term shoulder function was 40 (IQR 27–46). OSS was available for 19 of 31 (61%) patients who needed a reoperation and for 113 of 117 (97%) patients who did not undergo secondary surgery. Patients who did not require secondary surgery had a significantly better shoulder score than those who underwent a reoperation (median OSS (IQR) = 41 (33–46) versus 25 (13–32); *p* < 0.001).

## Discussion

We found that the use of calcar screws and adequate reduction of the fracture, to achieve medial support, significantly reduced the risk of a reoperation due to fixation failure in patients with a PHF treated with a locking plate*.*

Medial support can be restored by anatomical reduction in fractures without medial comminution to reduce the risk of fixation failure. In fractures with medial comminution, calcar screws, medialization and impaction of the humeral shaft onto the humeral head or both, may restore a medial support [10]. Alternatively, some authors advocate the use of an additional medial buttress plate [18], allograft reinforcement [19, 20], bone substitution [9, 21] or intramedullary nailing [22–24].

Although we were aware of the possible importance of calcar screws during the study period, calcar screws were only used in half of the patients. One possible explanation was that the less invasive deltoid split approach was introduced during the study period, and that calcar screws were initially avoided using this approach to prevent iatrogenic axillary nerve injury. However, with increased experience with the deltoid split approach, calcar screws could be placed safely [25]. A cadaveric study has shown improved stability in the fracture with calcar screws, both in fractures with medial comminution and in fractures with intact medial column, suggesting the use of calcar screws in all PHFs in an osteoporotic bone [26]. On the other side, calcar screws may not be sufficient to prevent fixation failure if the fracture is fixed in varus and may not be essential when there is good medial support [27].

In keeping with a previous study, we found that postoperative varus malalignment significantly increased the risk of a reoperation due to fixation failure [12]. Interestingly we did not find that preoperative varus was an independent risk factor for fixation failure, which contradicts the findings presented by Jung et al. [28]. However, both pre and postoperative varus malalignment have been associated with an increased risk of fixation failure [29]. Residual postoperative varus was more often found in patients who presented with a preoperative varus malalignment, and surgeons should be aware that such PHF subtypes may be especially at risk for fixation failure.

In the present retrospective cohort study, the number of reoperations (16%) was in the lower range of previously published data in two systematic review articles, ranging from 13 to 30% [5, 30]. Patients who underwent a reoperation reported poor long-term shoulder function, compared to good shoulder function reported by those who did not undergo a secondary surgery. The poorer outcomes following revision surgery are in line with other studies [31, 32], which points out the importance of a successful first intervention. The overall long-term shoulder function in our cohort was good, which is consistent with a multicentre randomised controlled trial that compared surgical treatment to conservative treatment in adult patients with PHF [33]. The authors of that study did not find any advantage of surgical over conservative management in terms of shoulder pain and function after 2 years. However, critics have questioned the recruitment process, as 1000 of 1250 (80%) eligible patients were not included, counting 87 patients with a “clear indication for surgery” [34].

Primary reverse total shoulder arthroplasty (RTSA) has been advocated to avoid revision surgery following inadequate reduction and unstable fixation [35]. RTSA has gained popularity and showed promising results, especially in elderly patients [36], also when compared to locking plate fixation [37]. However, RTSA complications are potentially adverse and include prosthetic joint infection, instability, neurological injury, scapular notching and periprosthetic fractures [38]. Still, RTSA may be a relevant choice for the primary surgical treatment of displaced PHFs among patients aged 65 years and above with poor rotator cuff status and high risk of healing complications [9, 39]. However, there are few level I trials, and two ongoing randomised controlled multicentre trials compare treatment of PHFs with locking plate versus RTSA [18] and locking plate versus RTSA or versus conservative treatment [40]. Hopefully, these trials will prove high-level evidence and guide the surgical treatment of proximal humeral fractures accordingly.

The main limitations of the current study were the retrospective design, possibly missing complications treated at other hospitals, and the lack of a matched control group to assess shoulder function in conservatively treated patients. Furthermore, we did not have radiographs of the contralateral uninjured shoulder, possibly introducing classification bias when evaluating the postoperative malalignment. The main strengths of our study were the number of patients and the high response rate for OSS questionnaires (89%).

## Conclusion

Medial support seems to be important to enhance stability and to avoid fixation failure in proximal humeral fractures and, hence, revision surgeries. In the present retrospective cohort study of 190 patients with a proximal humeral fracture treated with a locking plate, the use of calcar screws and the absence of postoperative varus malalignment (head-shaft angle > 120°), significantly reduced the risk of fixation failure. We, therefore, recommend the use of calcar screws and to avoid residual varus malalignment to improve the medial support of proximal humeral fractures treated with a locking plate.

## Abbreviations

AVN: Avascular necrosis
HSA: Head-shaft angle
ICC: Intraclass correlation coefficient
IRR: Inter-rater reliability
OSS: Oxford Shoulder Score
PHF: Proximal humeral fractures
RTSA: Reverse total shoulder arthroplasty

## References

[1] Lauritzen JB, Schwarz P, Lund B, McNair P, Transbol I (1993). Changing incidence and residual lifetime risk of common osteoporosis-related fractures. Osteoporos Int.

[2] Sumrein BO, Huttunen TT, Launonen AP, Berg HE, Fellander-Tsai L, Mattila VM (2017). Proximal humeral fractures in Sweden-a registry-based study. Osteoporos Int.

[3] Handoll HH, Brorson S (2015). Interventions for treating proximal humeral fractures in adults. Cochrane Database Syst Rev.

[4] Bell JE, Leung BC, Spratt KF, Koval KJ, Weinstein JD, Goodman DC, Tosteson AN (2011). Trends and variation in incidence, surgical treatment, and repeat surgery of proximal humeral fractures in the elderly. J Bone Joint Surg Am.

[5] Launonen AP, Lepola V, Flinkkila T, Laitinen M, Paavola M, Malmivaara A (2015). Treatment of proximal humerus fractures in the elderly: a systemic review of 409 patients. Acta Orthop.

[6] Kralinger F, Unger S, Wambacher M, Smekal V, Schmoelz W (2009). The medial periosteal hinge, a key structure in fractures of the proximal humerus: a biomechanical cadaver study of its mechanical properties. J Bone Joint Surg Br.

[7] Osterhoff G, Ossendorf C, Wanner GA, Simmen HP, Werner CM (2011). The calcar screw in angular stable plate fixation of proximal humeral fractures--a case study. J Orthop Surg Res.

[8] Yang P, Zhang Y, Liu J, Xiao J, Ma LM, Zhu CR (2015). Biomechanical effect of medial cortical support and medial screw support on locking plate fixation in proximal humeral fractures with a medial gap: a finite element analysis. Acta Orthop Traumatol Turc.

[9] Laux CJ, Grubhofer F, Werner CML, Simmen HP, Osterhoff G (2017). Current concepts in locking plate fixation of proximal humerus fractures. J Orthop Surg Res.

[10] Gardner MJ, Weil Y, Barker JU, Kelly BT, Helfet DL, Lorich DG (2007). The importance of medial support in locked plating of proximal humerus fractures. J Orthop Trauma.

[11] Agel J, Jones CB, Sanzone AG, Camuso M, Henley MB (2004). Treatment of proximal humeral fractures with Polarus nail fixation. J Shoulder Elb Surg.

[12] Agudelo J, Schurmann M, Stahel P, Helwig P, Morgan SJ, Zechel W, Bahrs C, Parekh A, Ziran B, Williams A (2007). Analysis of efficacy and failure in proximal humerus fractures treated with locking plates. J Orthop Trauma.

[13] Jeong J, Bryan J, Iannotti JP (2009). Effect of a variable prosthetic neck-shaft angle and the surgical technique on replication of normal humeral anatomy. J Bone Joint Surg Am.

[14] Marsh JL, Slongo TF, Agel J, Broderick JS, Creevey W, DeCoster TA, Prokuski L, Sirkin MS, Ziran B, Henley B (2007). Fracture and dislocation classification compendium - 2007: orthopaedic trauma association classification, database and outcomes committee. J Orthop Trauma.

[15] Ekeberg OM, Bautz-Holter E, Tveita EK, Keller A, Juel NG, Brox JI (2008). Agreement, reliability and validity in 3 shoulder questionnaires in patients with rotator cuff disease. BMC Musculoskelet Disord.

[16] Randsborg PH, Fuglesang HF, Rotterud JH, Hammer OL, Sivertsen EA (2014). Long-term patient-reported outcome after fractures of the clavicle in patients aged 10 to 18 years. J Pediatr Orthop.

[17] Cicchetti DV (1994). Guidelines, criteria, and rules of thumb for evaluating normed and standardized assessment instruments in psychology. Psychol Assess.

[18] Fjalestad T, Iversen P, Hole MO, Smedsrud M, Madsen JE (2014). Clinical investigation for displaced proximal humeral fractures in the elderly: a randomized study of two surgical treatments: reverse total prosthetic replacement versus angular stable plate Philos (the DELPHI-trial). BMC Musculoskelet Disord.

[19] Katthagen JC, Schwarze M, Meyer-Kobbe J, Voigt C, Hurschler C, Lill H (2014). Biomechanical effects of calcar screws and bone block augmentation on medial support in locked plating of proximal humeral fractures. Clin Biomech (Bristol, Avon).

[20] Hinds RM, Garner MR, Tran WH, Lazaro LE, Dines JS, Lorich DG (2015). Geriatric proximal humeral fracture patients show similar clinical outcomes to non-geriatric patients after osteosynthesis with endosteal fibular strut allograft augmentation. J Shoulder Elb Surg.

[21] Schliemann B, Wahnert D, Theisen C, Herbort M, Kosters C, Raschke MJ, Weimann A (2015). How to enhance the stability of locking plate fixation of proximal humerus fractures? An overview of current biomechanical and clinical data. Injury.

[22] Gadea F, Favard L, Boileau P, Cuny C, d'Ollone T, Saragaglia D, Sirveaux F (2016). Sofcot: fixation of 4-part fractures of the proximal humerus: can we identify radiological criteria that support locking plates or IM nailing? Comparative, retrospective study of 107 cases. Orthop Traumatol Surg Res.

[23] Kloub M, Holub K, Polakova S (2014). Nailing of three- and four-part fractures of the humeral head -- long-term results. Injury.

[24] Dilisio MF, Nowinski RJ, Hatzidakis AM, Fehringer EV (2016). Intramedullary nailing of the proximal humerus: evolution, technique, and results. J Shoulder Elb Surg.

[25] Gardner MJ, Boraiah S, Helfet DL, Lorich DG (2008). The anterolateral acromial approach for fractures of the proximal humerus. J Orthop Trauma.

[26] Ponce BA, Thompson KJ, Raghava P, Eberhardt AW, Tate JP, Volgas DA, Stannard JP (2013). The role of medial comminution and calcar restoration in varus collapse of proximal humeral fractures treated with locking plates. J Bone Joint Surg Am.

[27] Bai L, Fu Z, An S, Zhang P, Zhang D, Jiang B (2014). Effect of calcar screw use in surgical neck fractures of the proximal humerus with unstable medial support: a biomechanical study. J Orthop Trauma.

[28] Jung SW, Shim SB, Kim HM, Lee JH, Lim HS (2015). Factors that influence reduction loss in proximal humerus fracture surgery. J Orthop Trauma.

[29] Hardeman F, Bollars P, Donnelly M, Bellemans J, Nijs S (2012). Predictive factors for functional outcome and failure in angular stable osteosynthesis of the proximal humerus. Injury.

[30] Gupta AK, Harris JD, Erickson BJ, Abrams GD, Bruce B, McCormick F, Nicholson GP, Romeo AA (2015). Surgical management of complex proximal humerus fractures—a systematic review of 92 studies including 4500 patients. J Orthop Trauma.

[31] Jost B, Spross C, Grehn H, Gerber C (2013). Locking plate fixation of fractures of the proximal humerus: analysis of complications, revision strategies and outcome. J Shoulder Elb Surg.

[32] Kristensen MR, Rasmussen JV, Elmengaard B, Jensen SL, Olsen BS, Brorson S (2018). High risk for revision after shoulder arthroplasty for failed osteosynthesis of proximal humeral fractures. Acta Orthop.

[33] Rangan A, Handoll H, Brealey S, Jefferson L, Keding A, Martin BC, Goodchild L, Chuang LH, Hewitt C, Torgerson D (2015). Surgical vs nonsurgical treatment of adults with displaced fractures of the proximal humerus: the PROFHER randomized clinical trial. JAMA.

[34] Dean BJ, Jones LD, Palmer AJ, Macnair RD, Brewer PE, Jayadev C, Wheelton AN, Ball DE, Nandra RS, Aujla RS (2016). A review of current surgical practice in the operative treatment of proximal humeral fractures: does the PROFHER trial demonstrate a need for change?. Bone Joint Res.

[35] Cazeneuve JF, Cristofari DJ (2006). Grammont reversed prosthesis for acute complex fracture of the proximal humerus in an elderly population with 5 to 12 years follow-up. Rev Chir Orthop Reparatrice Appar Mot.

[36] Iacobellis C, Berizzi A, Biz C, Camporese A (2015). Treatment of proximal humeral fractures with reverse shoulder arthroplasty in elderly patients. Musculoskelet Surg.

[37] Giardella A, Ascione F, Mocchi M, Berlusconi M, Romano AM, Oliva F, Maradei L (2017). Reverse total shoulder versus angular stable plate treatment for proximal humeral fractures in over 65 years old patients. Muscles Ligaments Tendons J.

[38] Cheung E, Willis M, Walker M, Clark R, Frankle MA (2011). Complications in reverse total shoulder arthroplasty. J Am Acad Orthop Surg.

[39] Kancherla VK, Singh A, Anakwenze OA (2017). Management of acute proximal humeral fractures. J Am Acad Orthop Surg.

[40] Launonen AP, Lepola V, Flinkkila T, Strandberg N, Ojanpera J, Rissanen P, Malmivaara A, Mattila VM, Elo P, Viljakka T (2012). Conservative treatment, plate fixation, or prosthesis for proximal humeral fracture**.** A prospective randomized study. BMC Musculoskelet Disord.

